# A Case of Pneumonia Caused by Pasteurella multocida in an Immunocompetent Indian Male

**DOI:** 10.7759/cureus.28820

**Published:** 2022-09-06

**Authors:** Sankalp Yadav

**Affiliations:** 1 Medicine, Shri Madan Lal Khurana Chest Clinic, New Delhi, IND

**Keywords:** dog bites, pasturella multocida, covid-19, tuberculosis, pneumonia

## Abstract

*Pasteurella multocida* is a common cause of cutaneous and soft-tissue infections. Cases of primary diseases in the lung result from the inhalation of airborne contaminants from infected nasopharyngeal secretions from pets such as cats and dogs. Clinical presentations due to *P. multocida *vary from a cough with or without hemoptysis or chest pain to severe debility. This is common in elderly populations with existing pulmonary diseases such as chronic obstructive pulmonary disease and bronchiectasis. The present case highlights *P. multocida* pneumonia in an Indian zookeeper with a pet dog who had no underlying lung pathology. This case emphasizes the significance of a detailed history with a physical examination backed by an extensive laboratory workup as the clinical features of *P. multocida* pneumonia resemble several common bacterial and viral infections.

## Introduction

Zoonotic infections caused by *Pasteurella* species belonging to the Pasteurellae family are common [1]. Cutaneous and soft-tissue infections caused by *Pasteurella multocida* are widely reported in the literature [2]. These soft-tissue infections result when an infected animal bites or scratches [3]. *P. multocida* also has the potential to cause lung illness after inhalation of infected droplets from infected pets or other animals [2]. *P. multocida* is a gram-negative coccobacillus that colonizes the nasopharynx and gastrointestinal (GI) tract of many animals [2].

Although *P. multocida* infection is commonly reported in immunocompromised patients, reports in immunocompetent individuals are also available [4]. The cases with pulmonary infection are generally elderly patients with an underlying pulmonary illness like chronic obstructive pulmonary disease (COPD), bronchiectasis, or malignancy [5]. The disease can present as pneumonia, tracheobronchitis, lung abscess, and/or empyema [5]. *P. multocida* cases often share similarities in clinical features with several other pathogens [5]. Differential diagnosis involves ruling out tuberculosis (TB), late evolution of organizing pneumonia in coronavirus disease 2019 (COVID-19), non-tuberculous mycobacteriosis (NTM), and other pneumonia in endemic areas. It requires a high index of suspicion to rule out other common diseases which are indistinguishable from *P. multocida* to establish the diagnosis. Here, a case of pneumonia in a 41-year-old immunocompetent Indian male is presented with no skin or soft-tissue involvement and who had a history of exposure to animals at the zoo and had a pet dog.

## Case presentation

A 41-year-old, non-diabetic, immunocompetent Indian male working at the local zoo and owner of a pet dog reported to the outpatient department with chief complaints of cough with thick expectoration and breathlessness on walking and subjective fever without chills or rigors for three days.

He was well three days back when he developed a fever that was subjective with no diurnal variations and was relieved after taking over-the-counter antipyretic paracetamol. He also had a cough with thick productive expectoration. There was no hemoptysis. He had dyspnea on exertion which subsided on rest.

His medical and surgical history was unremarkable. There was no history of TB or COVID-19 in the family. He was a non-smoker and had no history of substance abuse. Moreover, there was no personal or family history of similar complaints. Additionally, there was no history of any travel to foreign countries or COVID-19 containment zones. He was a non-immigrant with no history of unemployment or contact with commercial sex workers or drug dealers.

**Figure 1:**
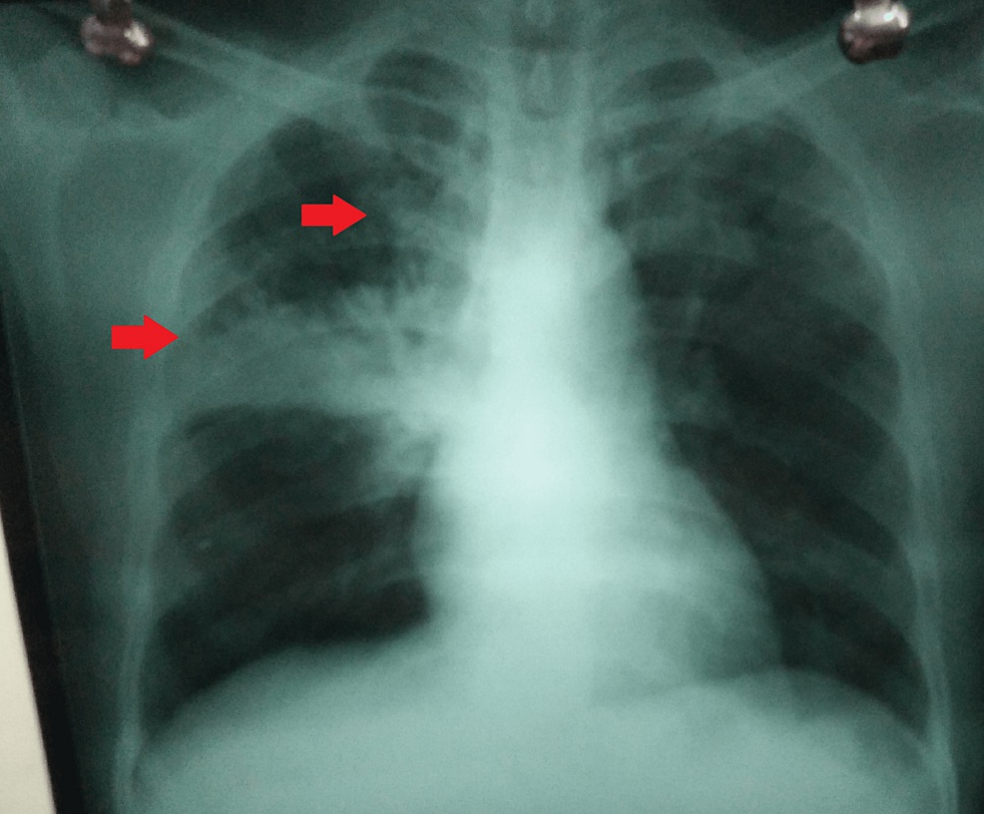
Chest radiograph posteroanterior view.

On examination, he was afebrile with a pulse of 79 beats/minute, blood pressure of 120/78 mmHg, tachypneic with a respiratory rate of 24 breaths/minute, and SpO_2_ of 93% on room air. His SpO_2_ fell to 90% on walking and returned to 93% on rest. He was fully conscious and oriented to time, place, and person with a Glasgow Coma Scale (GCS) score of 15/15. Physical examination revealed diminished breath sounds in the right lung with scattered crackles. There were no bite marks, scratches, or other skin findings, and there were no enlarged lymph nodes. The rest of the systemic examination was within normal limits. A provisional diagnosis of pneumonia was made, and a detailed lab workup with a chest radiograph was advised. The lab data was remarkable for an increased leukocyte count of 13.1 K/µL, C-reactive protein of 91.3 mg/L, and procalcitonin of 15 ng/mL. A chest radiograph (posteroanterior) view was suggestive of right upper and middle lobe consolidation (Figure 1). A sputum sample was sent for Ziehl-Neelsen (ZN) for acid-fast bacillus (AFB) and cartridge-based nucleic acid amplification test, which was negative for* Mycobacterium tuberculosis*. An on-spot rapid antigen test and a reverse transcription-polymerase chain reaction for COVID-19 were negative. Human immunodeficiency virus I and II were non-reactive. Bronchoalveolar lavage (BAL) fluid cultures on Mueller-Hinton agar were suggestive of *P. multocida* which was sensitive to penicillin. Further, NTM was ruled out by smears for AFB, cultures, and polymerase chain reaction of two sputum specimens and BAL fluid. His blood culture was normal. Computed tomography of the chest was not done due to the non-availability of free spots and the patient’s refusal to bear the expenses.

Finally, he was diagnosed with a case of pneumonia caused by *P. multocida *and was started on treatment with amoxicillin-clavulanate for two weeks, following which his symptoms promptly reduced. He was advised to follow up in the respiratory medicine outpatient department but he never reported again.

## Discussion

*P. multocida* is a highly versatile, ubiquitous, opportunistic commensal belonging to the *Pasteurella* species and is found universally in domestic and wild animals [6]. It is small, non-flagellated, pleomorphic, and one of the most common known causes of zoonosis [6]. The high carriage rates of *P. multocida* are seen in domestic animals such as dogs and cats at 20-50% and 70-90%, respectively [2].

*P. multocida* is usually associated with chronic as well as acute infections that can cause considerable morbidity and mortality [7]. In humans, elderly and immunocompromised patients are vulnerable where cutaneous and soft-tissue involvement after animal bites or scratches are reported [8]. This bacteria is known to affect patients with an underlying lung pathology, especially in the upper and lower respiratory tracts [8,9]. It has also been reported to cause meningitis, peritonitis, septic arthritis, sepsis, and pneumonia [10].

The clinical features of respiratory tract infections caused by *P. multocida* are imprecise [7]. The disease onset can be gradual or abrupt [5,11]. The majority of patients report fever, dyspnea, malaise, and pleuritic chest pain [5,11]. However, the diagnosis of *P. multocida* is challenging, especially in a setting where other bacterial and viral infections are endemic. In countries where *M. tuberculosis* is prevalent, it requires a very detailed clinical examination with lab workup to diagnose *P. multocida*. NTM is also common and thus diagnostic bronchoscopy to determine the exact cause of infection is crucial. In this case, a diagnosis was established only after a BAL culture.

A case similar to this was reported by Itoh and Kurai where the diagnosis was made after BAL culture [7]. However, the present case differs from their case in the absence of any remarkable history of NTM infections. However, the present case shares similarities regarding contact with pets and wild animals at the zoo.

Another similar case was reported by Ayas et al. which required a great deal of laboratory workup due to the presentation during the COVID-19 pandemic [12]. This case shares similarities with their case of being diagnosed during the COVID-19 pandemic and certain clinical features such as shortness of breath. However, the present case differs in the absence of hemoptysis, diarrhea, and chest pain. Additionally, there was no history of atrial fibrillation, congestive heart failure, and hypertension.

To summarize, this is a case of an immunocompetent Indian zookeeper with a pet dog who presented with clinical features similar to some of the common infections prevalent in countries like India. After a detailed history and laboratory investigations, he was diagnosed with *P. multocida* pneumonia which was managed with a 14-day course of antibiotics.

## Conclusions

Animal handlers, such as zoo and farm workers, veterinarians, butchers, and animal breeders, and individuals with pets, such as dogs and cats, are always at risk of opportunistic zoonosis such as *P. multocida *pneumonia. The similarities of clinical features with other common infections including TB, COVID-19 (late phases of evolution and complications such as organizing pneumonia), or infections with NTM require a very high index of suspicion to establish the definitive diagnosis. BAL is very important in the determination of the exact cause. Moreover, if not treated, it can result in life-threatening outcomes. Therefore, it is imperative to include the workup for the diagnosis of *P. multocida* pneumonia in countries with a high burden of TB and COVID-19.
